# Statistical approaches for assessing meat quality and heifer rumen histology based on dietary forage

**DOI:** 10.3389/fvets.2024.1416365

**Published:** 2024-08-07

**Authors:** Alberto Benito-Díaz, Ainhoa Sarmiento-García, Juan José García-García, Ceferina Vieira, Esperanza Domínguez, Raúl Bodas Rodríguez, Luis Gómez-Gordo, Purificación Vicente-Galindo

**Affiliations:** ^1^Línea de Investigación en Rumiantes, Instituto Tecnológico Agrario de Castilla y León, Valladolid, Spain; ^2^Área de Producción Animal, Departamento de Construcción y Agronomía, Facultad de Agricultura y Ciencias Ambientales, Universidad de Salamanca, Salamanca, Spain; ^3^Estación Tecnológica de la Carne, Instituto Tecnológico Agrario de Castilla y León (ITACyL), Salamanca, Spain; ^4^Departamento de Anatomía y Anatomía Patológica Comparadas, Facultad de Veterinaria, Universidad de Extremadura, Cáceres, Spain; ^5^Departamento de Estadística, Facultad de Medicina, Universidad de Salamanca, Salamanca, Spain

**Keywords:** biplot, forage diet, heifer, meat quality, principal component analysis, ruminal histology

## Abstract

**Introduction:**

Feeding local forages to ruminants is a promising strategy for enhancing metabolic processes, promoting sustainable farming, and improving product quality. However, studies comparing the effects of different forages on rumen histology and meat attributes of heifers are limited and variable.

**Material and methods:**

This study evaluated the benefits of incorporating local forages into heifer diets by comparing barley straw (BS) and oat hay (OH) on heifer attributes focusing on meat quality (MQ) and rumen status (RS). Sixteen crossbred (Charolais x Limousin) female heifers (7 months of age, 263 ± 10.50 kg) were randomly assigned to two dietary treatments (BS or OH) over 120 days.

**Results and discussion:**

Heifers fed OH showed enhanced RS (*p* < 0.05), characterized by improved intestinal epithelial integrity and a lower percentage of hyperpigmented cells, suggesting a potential reduction in inflammatory processes compared to BS, which may indicate a lower risk of metabolic diseases. Despite this, no significant differences (*p* > 0.05) were found in animal performance, chemical composition, and technological properties of the meat between the dietary groups, while lower levels (*p* < 0.05) of certain saturated fatty acids (C12:0, C15:0, and C22:0) were found in the meat from heifers fed OH. Principal component analysis (PCA) reduced the variables and demonstrated that all variables assessed can be condensed into four new variables explaining 75.06% of the variability. Moreover, biplot analysis reveals that the OH diet could be discriminated from BS. Our findings suggest that OH is a valuable fiber source, positively influencing certain heifer attributes, and supporting sustainable animal agriculture practices.

## 1 Introduction

Since the world population is expected to reach 9.7 billion by 2050, developing strategies to feed this growing population, particularly those aimed at increasing protein production, is of, is of great concern (1, 2). Livestock production, especially ruminant animals, offers a potential solution to this challenge (3). Red meat, including beef and lamb, is a significant source of high-quality protein and essential nutrients necessary for a balanced diet (4, 5). The dietary contribution of meat is difficult to replace with other ingredients. Studies have shown that populations with limited access to meat often suffer from common health problems, including impaired growth in children, which meat consumption can help prevent (3, 5).

Ruminant production is predominantly conducted in intensive systems, which are frequently criticized for their ethical and environmental impacts (3). These production systems typically involve feeding animals diets high in concentrates and low in roughage to maximize weight gain and feed efficiency (5). However, this feeding strategy poses several risks. High starch intake from concentrates enhances ruminal microbial fermentation and the production of volatile fatty acids, primarily short-chain fatty acids (6). Reducing dietary forage in favor of concentrates results in less chewing and a significant decrease in ruminating time. This reduction in ruminating time lowers saliva production, which is essential for buffering the rumen and maintaining stable ruminal pH. It disrupts the ruminal activity causing difficulties in maintaining stable ruminal pH (4), eventually leading to ruminal acidosis (6).

Acute and subacute ruminal acidosis (SARA) are among the most significant metabolic disorders affecting beef and dairy cattle (7, 8). SARA appears to be a “silent disease” characterized by late symptoms (such as diarrhea, weight loss, poor body condition, or decrease in feed intake) making its prevention difficult (6). These symptoms are related to histological changes in the rumen epithelia, involving the destruction of ruminal papillae and the appearance of inflammatory phenomena (7–10). This process negatively affects the health status of the animals and economic profitability (6, 8, 11, 12). Adequate diet balancing, especially the forage component, is essential to avoid SARA incidence in the livestock herd (11, 13). The inclusion of forage in the diet of ruminants could reduce ruminal fermentation, improve rumen epithelium status, favor the salivation process, and slow down the pH decrease (4), enhancing performance parameters (11). Intake of forage leads to healthier meat by increasing the concentration of monounsaturated fatty acids (MUFA) and n-6 and decreasing the concentration of n-3 (11, 14). It has also been suggested that the intake of forages by ruminants could modify the color, lipid oxidation, or water retention capacity of meat as compared to conventional diets (11).

Oats (*Avena sativa L*), produced annually over 25 million Tn worldwide eare one of the key sources of ruminant feed, widespread in the Mediterranean region. This crop has remarkable socio-economic importance for medium and small farms due to its easy management and relatively low cost (13). Oat is considered a high-quality forage due to its content of protein, acid-detergent fiber, vitamins, and minerals (15). Hence, including oat hay (OH) in ruminant diets could be beneficial as a local economy-friendly forage (16, 17). Most previous studies have been focused on comparing the effect of forages versus concentrate on performance. However, studies comparing forages of different qualities on rumen histology and meat attributes (e.g., aromatic compounds, fatty acids) of heifers are scarce and show great variability (10). Since many variables of rumen histology and meat quality could be related to diet and forage quality, principal component analysis (PCA) seems promising to elucidate these relationships and reduce the variables tested. PCA is suitable for identifying from a pool of initial variables only those that affect the final result clearly and objectively, eliminating duplicated information (18). Representing the variables and individuals using a biplot diagram allows for a correct interpretation of the results (19). Thus, the current research aimed to compare the effects of including OH as an autochthonous crop compared with barley straw in the beef heifers’ diet on ruminal histology and meat quality and identify the predominant variables that have been affected by diet. This study offers a reference for promoting local forage production and may contribute to the circular economy by obtaining a sustainable farming system.

## 2 Materials and methods

### 2.1 Experimental design

The current research was evaluated and approved by the Animal Experimentation Service of the Instituto Tecnológico Agrario de Castilla y León, complying with the standards established by the Confederación de Sociedades Científicas de España (COSCE). Moreover, the criteria established by the European policy regarding the protection of animals were applied in the experimental period (18) and the ARRIVE 2.0 guidelines were also respected (20).

Sixteen crossbred (Charolais × Limousin) female heifers (*Bos taurus*) with similar weight (263 ± 10.5 kg of initial BW) and 210 days of age were purchased from the same commercial farm and included in the current research. Animals were raised at a local farm in Salamanca, Castilla y León, Spain (40° 49′ 34′, ‘5° 56’ 34″) for 120 days. After its arrival, heifer calves were randomly assigned to two experimental diets in a completely randomized design. Each experimental group consisted of two replicate groups, with four females per hutch. All the pens had the same dimensions (12.5 m^2^/pen) and environmental conditions and were well-aired, clean, and sanitized. Sawdust was used as bedding material in the pens. The adjoining pens were spaced with a metal mesh barrier that enabled animals’ visual contact with each other. All hutches were fitted with an automatic feeder to dispense feed and water *ad libitum*. Every day, individual heifer was checked to detect any disease signs.

All animals received a diet consisting of a cereal-based concentrate as well as mineral supplements presented in pellet form which was provided by a local company (Deheus Animal Nutrition, A Coruña, España) and supplemented with fiber sources. The experimental groups only differed in the source of fiber used. Group BS received barley straw (*Hordeum vulgare L*.), while group OH received oat hay (*Avena sativa L.*). Barley straw was provided by a local company (Salamanca, Castilla y León, España), while the oat hay was collected at the same facilities where the experiment was conducted. Forages were chopped before being offered to the animals. The diets were balanced to be isoproteic (14.20%) and isoenergetic (1340.00 kcal/kg), so, depending on the group, the proportion of forage included was different. In this sense, the concentrate: fiber ratio was adjusted to represent 91.20:8.80 for the BS group and 90:10 for the OH group (expressed as fresh matter). Each day the proper ratio of concentrate to forage was manually mixed before feeding to the animals. The concentrate was supplied daily in the feed bunkers with a separate bunker for forage, with no restrictions. The diets were formulated to satisfy the nutrient needs of heifer calves as provided by the National Academies of Sciences, Engineering, and Medicine (21).

The procedures described by AOAC (22) were followed to determine the chemical composition of both fiber sources and diets. The moisture content (2001.12) was obtained by drying at 105°C in an oven, the protein content was measured by Kjeldahl (990.03), fat was recovered from the samples in a Soxhlet with petroleum ether (2003.06) ash was determined by incineration and drying the water (942.05), while neutral detergent fiber (NDF) was determined using Van Soest Method. Table 1 presents the chemical composition and the nutritional value of both sources and the chemical composition of the diet (including fiber source and concentrate).

**Table 1:** Chemical composition of fiber sources and experimental diets.

	Fiber source		Experimental diet	
Chemical composition (g/kg)	Barley straw	Oat hay	BS group	OH group
Metabolizable energy (kcal/kg ME)	1200.00	1350.00	1340.00	1342.00
Dry matter	91.70	96.50	89.00	90.00
Crude protein	3.70	5.70	13.80	13.80
Crude fat	1.60	2.73	4.80	4.80
Crude fiber	36.00	38.90	9.10	9.10
Neutral detergent fiber (NDF)	72.00	65.40	23.90	24.20
Starch	0.70	5.10	42.40	42.40
Ash	7.20	3.57	76.00	76.00
Calcium	0.30	0.21	1.00	1.00
Available phosphorus	0.07	0.19	0.40	0.40

BS, Barley Straw; OH, Oat hay.

### 2.2 Postmortem sampling

#### 2.2.1 Histology processing

Carcasses from animals involved in the study were tracked from the point of slaughter until evisceration, leaving a delay between sacrifice and sample collection of approximately 10 min. An incision was made in the rumen by abattoir staff, which was completely hand-emptying and fully observed. A sample of about 8 × 4 cm was collected from the middle of the dorsal sac (*n* = 16) in a sterile tube. Then, it was transferred to the laboratory of Anatomía Patológica (Facultad de Veterinaria, Cáceres, Extremadura, España) in an isotherm vehicle to be processed.

Samples were washed in water to eliminate excess intake and fixed with 3.5% neutral (pH 7.2) buffer formaldehyde solution (0.1 M) for 48 h. Then, the tissue samples were dehydrated using ascending alcohol scales (ethanol and xylene) and individually embedded in paraffin wax. Histological sections (5 μm) were cut on a Leica RM2255 microtome (Leica Biosystem, Barcelona, Sapin) and placed on slides (2 per sample). The sections were stained with hematoxylin–eosin solution for histological analysis. Firstly, all slides were scanned with a Nikon DS-R12 microscope (Nikon, Tokyo, Japan) equipped with a camera (DXMI200F, Nikon, Japan) connected to a computer using NIS-Elements L image analysis software (Nikon, Tokyo, Japan) that allowed capturing images of the state of the samples. The images were examined, ensuring that they were as complete as possible and that the field was free of artifacts, for subsequent measurements, with each image being taken several times to ensure objectivity. All slides were examined and scored by an operator to ensure uniformity of scores, except for the reduction of rumen papillae, for evaluating ruminal histology, continuous variables, and ordinal categorical variables. Ordinal categorical variables were assessed using a scoring system similar to the one described by Ferguson et al. (10). The integrity of epithelium, the presence of basophilic or hyperpigmented cells, and the abundance of perinuclear vacuolation were considered standards of ruminal damage. Table 2 summarizes the parameters and the criteria employed for ruminal evaluation.

**Table 2:** Overview of characteristics associated with a standardized histological punctuation system for heifer rumen.

Name	Variable	Definition and measurement	Levels	Level definitions
Ruminal papillae thickness (μm)	Continuous	The mean of 15 measurements from the distance between the base and the tip of the papillae at approximately 500 μm from the apical edge (using ×100 magnification)	NA	NA
Reduction of rumen papilla height (%)	Continuous	It was based on the experiences of two investigators, which were studied separately (using ×40 magnification). Data were obtained using an interval with a variation of 10%. The value was considered adjusted if both investigators agreed on the assessment; if the difference was greater than 10%, the sample was studied again and discussed	NA	NA
Lamina propria thickness (%)	Continuous	The mean of 15 measurements of lamina propria (μm) taking the presence of smooth muscle fibers along the longitudinal axis as orientation (using ×100 magnification). Then it was expressed as a percentage.	NA	NA
Epithelium thickness (%)	Continuous	To minimize accuracy, the thickness of the epithelium was calculated from the measurement of the lamina propria.	NA	NA
Integrity of the epithelium	Ordinal categorical	This parameter was evaluated according to two characteristics: Presence of clefts, buds, and branches along the papillae, using x40 magnification The extent to which the epithelium forms a complete, uninterrupted layer over the papillae, using ×100 magnification	1–3	1- All or almost all papillae are simple, without clefts, outgrowths, or branching, and show intact epithelium or less than 25%. 2- A minority of papillae show some degree of clefting, budding, or branching, and show some alteration in the integrity of the epithelium (26–50% affected). 3- Most papillae show some degree of clefting, budding, or branching, with clear disruptions in the integrity of the epithelium (more than 50% affected).
Region of thickening	Ordinal categorical	Once the samples with rumen damage have been identified, localization of the thickening region using ×40 magnification	1–3	1- Epithelium 2- Lamina propria 3- Both tissues
Presence of basophilic/hyperpigmented cells	Ordinal categorical	The presence of hyperpigmented cells in any papillae observed using ×100 magnification	0–3	0 – No basophilic cells seen 1 – Few or occasional basophilic cells seen (1–33% presence) 2- Certain basophilic cells seen (34–66%) 3- The majority of cells present basophilic cytoplasm (>66%)
Perinuclear vacuolation	Ordinal categorical	Presence of perinuclear vacuoles (foaming cells) in the using ×100 magnification	0,1	0 – No vacuolated cells seen 1 – Few or occasional vacuolated cells seen (1–33% presence) 2- Certain vacuolated cells seen (34–66%) 3- The majority of cells present vacuoles (>66%)

NA, not applicable.

#### 2.2.2 Carcass traits

After 24 h of carcass chilling at 4 C, meat samples (*n* = 16) were collected from the *M. longissimus thoracis* (MLT) between the 9th and 11th rib on the right carcass side, which was previously weighed (kg) and recorded. The samples were transferred under refrigerated conditions (4 ± 1°C) and stored for 24 h at the Estación Tecnológica de la Carne (Guijuelo, Salamanca, Spain) for analysis. Then, the subcutaneous fat and epimysium were removed, and MLT was divided into two portions: the proximal portion was used for analyzing chemical composition, pH, and color, while the distal portion was used for analyzing volatile compounds.

##### 2.2.2.1 Technological properties of the meat

The pH and color were measured after 24 h post-mortem in the laboratory. Samples were taken from the refrigerator (4 ± 1°C), and the pH was measured in triplicate using a pre-calibrated Crison pH Meter BASIC 20^®^ (Hach Lange Spain, L’Hospitalet de Llobregat, Barcelona, Spain) equipped with a penetration electrode. To determine color, the surface of the proximal portion was left at room temperature in the dark to bloom for 90 min. Immediately thereafter, the instrumental evaluation of color was conducted three times at different locations on the sample using a Minolta 2006d spectrophotometer in the CIE L* a* b* space with D65 illumination, a 10° observer visual angle and SCI mode conditions as described by Vieira et al. (21).

##### 2.2.2.2 Proximal composition and intramuscular fat composition

The proximal portion of the samples was ground up to determine the proximate chemical composition according to the AOAC method (22) (including dry matter, crude protein, and crude fat). The intramuscular fat was extracted using the di the method proposed by Folch et al. (23), and then, fatty acid methyl esters were obtained using identical conditions to those described by Vieira et al. (24). Subsequently, an Agilent Technologies 6,890 gas chromatograph (GC) (Santa Clara, CA, USA) was used for determining fatty acid composition. The content of fatty acids was estimated by identifying the peak areas of the chromatograms and comparing their retention time with those of commercially available reference standards (FAME mix, Sigma). Then, they were expressed as g per 100 g of the total amount of fatty acid methyl esters identified. Furthermore, the percentage of saturated fatty acids (SFA), monounsaturated fatty acids (MUFA), polyunsaturated fatty acids (PUFA), and n-6/n-3 ratio were determined as reported by Vieira et al. (24).

##### 2.2.2.3 Volatile compounds

The volatile compounds were assessed in duplicate using the SPME-gas chromatography-mass–mass spectrometry, following the procedure described by Vieira et al. (25). The procedure was carried out in duplicate. The distal section of each MLT was cut into 2 cm thick slices. These slices were grilled on a double-sided griddle preheated to 220°C for 10 min until they reached a core temperature of 70°C. Subsequently, two grams of the cooked meat were mixed with 4.7 mL of water and 0.07 g of NaCl, homogenized, and placed into 20 mL headspace vials. The vials were then sealed with magnetic screw caps (Agilent Technologies, Santa Clara, CA, USA), and allocated in a CG 7890A (Agilent Technologies, Santa Clara, FL, USA) connected to an MSD 5975 C mass spectrometer (Agilent Technologies), fitted with a CTC Combi PAL autosampler (CTC Analytics AG, Zwingen, Switzerland) for the determination of volatile compounds.

Volatile compounds were segregated using a DB-5MS column (60 m × 0.25 mm ID × 0.25 μm film thickness, J&W Scientific, Folsom, CA, USA). Helium had been employed with a steady rate of 1.5 mL/min as the carrier gas. Before injecting, each vial was balanced at 60°C on a mixer under continuous stirring (750 rpm, 5 s on, 2 s off) over 40 min. Then 1 mL of the headspace gas was pumped onto the chromatograph with a 2.5-mL CombiPAL headspace syringe (CTC Analytics AG). The syringe needle temperature was set at 100°C, while the velocities of injection and filling were 100 μL/s and 250 μL/s. The injector temperature was kept at 260°C in a divided injection way at a 2:1 split ratio. The temperature program of the oven consists of an opening step at 35°C for 1 min, continued with linear increments from 35 to 50°C at 10°C/min, then to 200°C at 4°C/min, and to 250°C at 50°C/min, ending this temperature for 11 min. The mass spectrometer was run in electron impact operation mode with an electron energy of 70 eV and an output current of 50 μA and scanned from 40 to 350 m/z at 3.94 scans/s.

Tentative identification of volatile compounds was carried out by comparing the mass spectra of the identified volatile compounds with those obtained in the NIST/EPA/NIH-98 Mass Spectral Database. The linear retention indexes were estimated using and comparing their linear retention indices, which were calculated from the retention times of a series of n-alkanes (Hydrocarbons/C5–C20; Sigma-Aldrich, St. Louis, MO, USA), with those found in the literature (21).

### 2.3 Statistical analysis

R 4.0.2 software program, has been used to analyze the variance of the data, considering the animal as the test unit. Meat quality and continuous variables of histological analysis were analyzed using one-way ANOVA, while ordinal categorical variables of ruminal status were evaluated using the chi-square test. A Tukey’s HSD means separation test was applied for *post hoc* analysis (*α* = 0.05). Continuous variables from histological analysis and meat quality results were expressed as mean ± standard errors of the mean (SEM) and the ordinal categories from the histological analysis were expressed as the percentage of cases present (0,1,2,3) for each of the diets studied. Statistical significance was assumed as a probability value of *p* < 0.05, while a probability value of *p* < 0.10 was defined as a trend.

PCA was applied as a multivariate statistical approach, to assess the relationship between dietary treatments and ruminal status or meat quality. PCA was applied to produce biplots displaying correlations that summarize the associations among variables applying an orthogonal processing to transform variables that are potentially related to each other into a collection of non-correlated variables known as principal components (PC).

## 3 Results

### 3.1 Histology

#### 3.1.1 Continuous variables and ordinal categorical data of ruminal histology

Findings of histological tests of ruminal slides described from all heifers means and standard error means of all continuous variables are provided in Table 3. No differences (*p* > 0.05) were described for any of the parameters studied. However, it is important to note that the reduction in papillae height in the BS group was twice that in the OH group, although those differences were not significant (*p* > 0.05).

**Table 3:** Continuous variables measured in the rumen of heifers (*n* = 16) fed with two experimental diets.

Continuous variables	BS group	OH group	*p*-value
Ruminal papillae thickness (μm)	332.40 ± 52.696	312.10 ± 50.149	0.443
Reduction of rumen papilla height (%)	61.25 ± 32.266	33.75 ± 32.486	0.112
Lamina propria thickness (%)	42.11 ± 9.565	47.33 ± 10.456	0.316
Epithelium thickness (%)	57.89 ± 9.565	52.68 ± 10.456	0.316

BS, Barley Straw; OH, Oat hay.

Ordinal categorical data about the ruminal health status of heifers are presented in Table 4. As can be observed, significant differences were reported for the parameters assessed. Regarding the integrity of epithelium, differences (*p* < 0.05) were observed depending on the diet received. The animals that received the BS diet exhibited a greater proportion of ruminal epithelium damage (in grades 2 and 3) as compared to those fed with OH in the diet. The loss of differentiated cellular structure in the epithelium was more evident in the BS group animals, as well as a greater presence of clefts in the papillae compared to the OH group. Regarding the region of thickening, differences were described depending on the tissue affected (*p* < 0.01). In the samples that had been identified as damaged tissue, it was noted that the heifer receiving dietary BS showed thickening which affected the epithelium (25%), the lamina propria (25%), and both tissues (18.75%), according to the percentages indicated. This is in contrast with those observed in the affected samples of the animals fed OH, where both tissues were thickening. The abundance of hyperpigmented cells and perinuclear vacuolation was considered a measure of the degree of ruminal damage. Concerning the count of hyperpigmented cells, a significantly different distribution (*p* < 0.01) was observed depending on the experimental group assessed. Heifers in the BS group exhibited a higher percentage (25.00% vs. 6.25%) of samples classified as grade 3 compared to OH group heifers. This indicates an increased percentage of cells with basophilic cytoplasm (>66%). Similar findings were noted for perinuclear vacuolation, where a greater number of samples were classified as grade 3 (indicating a significant presence of vacuoles within the cell) in heifers fed BS compared to those fed OH (12.50% vs. 6.25%). However, despite these numerical differences, statistical analysis revealed that they were not statistically significant (*p* > 0.05).

**Table 4:** Ordinal categorical data about the ruminal integrity of heifers (*n* = 16) fed two different diets expressed as distribution percentages.

	BS group				OH group				
Ordinal categorical varaibles	0	1	2	3	0	1	2	3	*p*-value
Integrity of the epithelium	–	0.00	25.00	18.75	–	25.00	18.75	6.25	0.017
Region of thickening		25.00	6.25	18.75	–	0.00	0.00	50.00	0.006
Presence of hyperpigmented cells	0.00	6.25	18.75	25.00	18.75	6.25	18.75	6.25	0.037
Perinuclear vacuolation	12.50	12.50	12.50	12.50	18.75	6.25	18.75	6.25	0.678

BS, Barley Straw; OH, Oat hay Categorical data are expressed as the presence in the percentage of each study factor. Higher scores (0–3) are related to a greater degree of ruminal damage, except for the thickening region. In this case, the number refers to the area of damage; 1- Epithelium, 2- Lamina propria, 3- Both tissues.

#### 3.1.2 Tissue architecture findings

**Figure 1:**
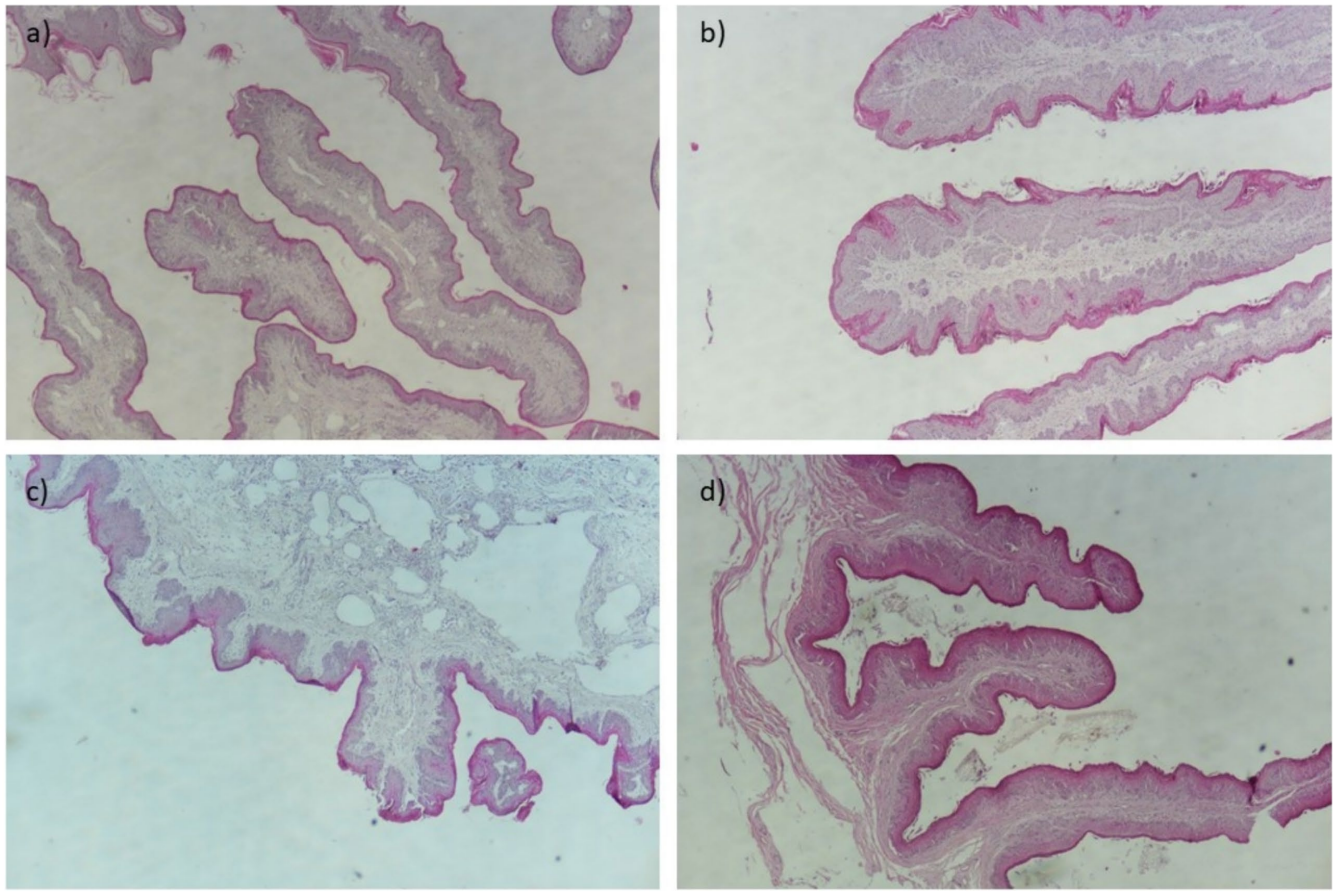
Representative cross-sections of ruminal papilla damage at low magnification (×40). **(A)** Affected ruminal papilla showing thickening of the lamina propria (2). **(B)** Affected ruminal papilla exhibiting thickening of the epithelium (1). **(C)** Loss of integrity of the papilla with thickening of the lamina propria (2) at low magnification. **(D)** Reduction in height of rumen papilla.

**Figure 2:**
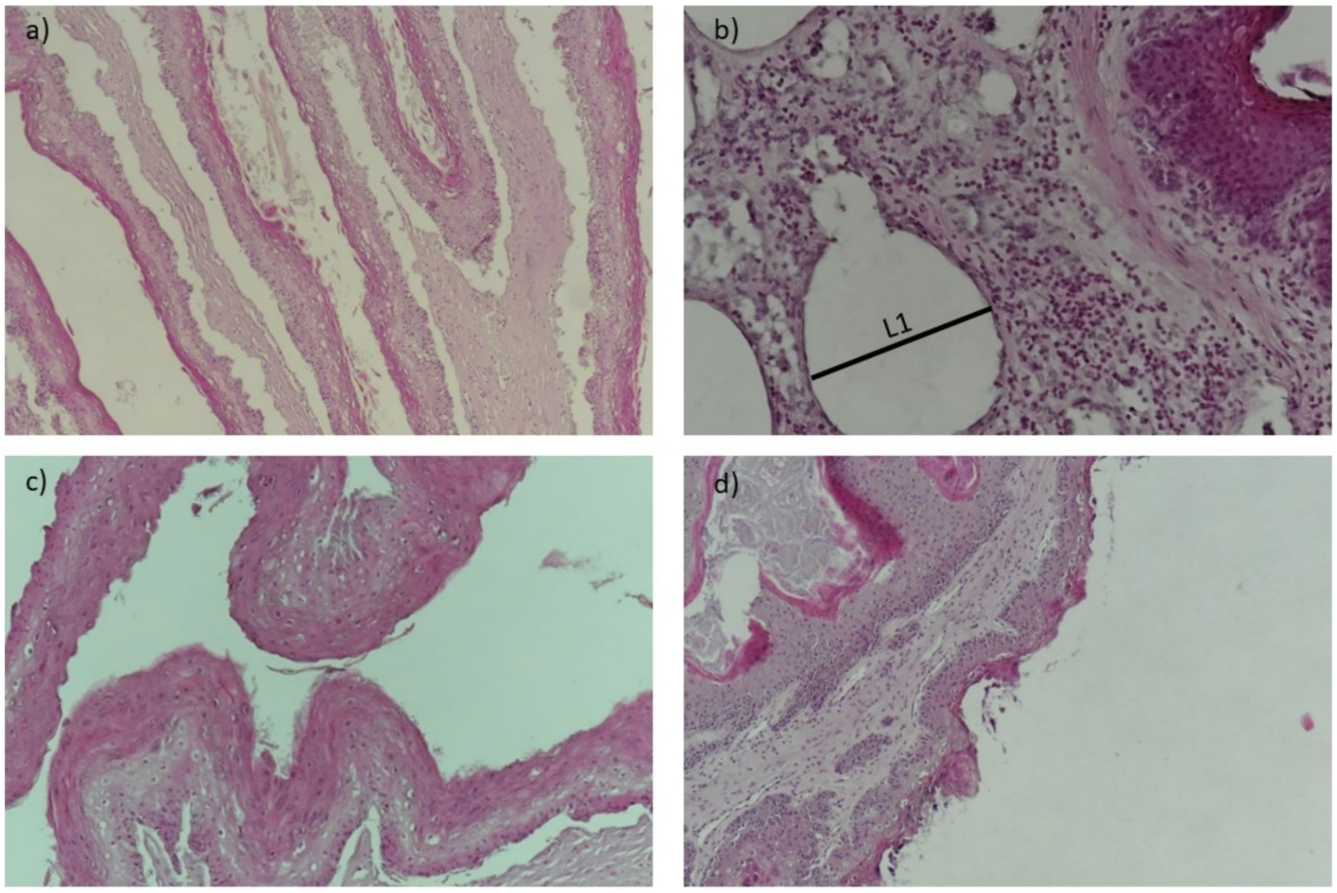
A representative cross-section of the loss of ruminal integrity. **(A)** Clear disruptions in the integrity of the epithelium at low magnification (×40). **(B)** Increase of vessel diameter (L1) and presence of eosinophilic content (×200). **(C)** High presence of perinuclear vacuoles (foaming cells) at high ×100 magnification. **(D)** Elevated incidence of cells with basophilic cytoplasm and cells containing vacuoles (×100).

The visual results of the categorical and continuous variables are presented in Figures 1, 2. Figure 1 depicts representative sections of damaged ruminal papillae stained with hematoxylin–eosin at low magnification (40×), with annotations highlighting distinct histological changes including thickened lamina propria and epithelium. These annotations are intended to elucidate the precise anatomical alterations observed in the tissue samples, facilitating a clearer understanding for readers. Figure 2 illustrates a representative cross-section demonstrating the loss of ruminal integrity. Figure 2A highlights clear disruption in the integrity of the epithelium at low magnification (×40). Figures 2B–D provide detailed views at higher magnifications (×200 and ×100) showing specific features: an increase in vessel diameter (L1) and presence of eosinophilic content, high presence of perinuclear vacuoles (foaming cells), and an elevated incidence of cells with basophilic cytoplasm and vacuolated cells, respectively. These findings in Figure 2 underscore the varied nature and severity of ruminal papilla damage observed in our study, providing critical insights into the histopathological changes associated with dietary intervention.

### 3.2 Assessment of meat quality

At the end of the experiment, all animals remained alive, with no signs compatible with pathological processes, and no veterinary treatment was necessary during the whole process. Table 5 includes the results obtained for the final weight of the animals and the MLT, as well as the chemical composition, technological properties, and fatty acid profile of the MLT of both experimental groups. Interestingly, no statistical differences (*p* > 0.05) in terms of final body weight and MLT weight of heifers between both experimental diets were found. Regarding the proximal composition of MLT, the values obtained were similar (*p* > 0.05) for both experimental groups. Color, which was described as lightness index (L*), red (a*), and yellow (b*), did not show statistical differences (*p* > 0.05) between the two treatments. Similarly, the pH value was not influenced by dietary treatment (*p* > 0.05). Regarding the fatty acid profile (Table 5), the most abundant fatty acids in MLT were palmitic acid (C16:0) as SFA, oleic acid (C18:1 n-9) as MUFA, and arachidonic acid (C20:4 n-6) as PUFA. The concentration of lauric (C12:0), pentadecanoic (C15:0), and docosanoic acid (C22:0) was significantly higher (*p* < 0.05) in the samples of the BS group compared to those obtained in the OH group. A similar trend (*p* < 0.1) was observed for myristic acid (C14:0), which increased in the BS group. The rest of the fatty acids evaluated in the MLT presented similar values in the two experimental diets. No differences were observed in the total fatty acid fractions (SFA, PUFA, and MUFA) examined or in the n-3 and n-6 content.

**Table 5:** Carcass traits, proximal composition, technological properties and fatty acid profile of the *M. longissimus thoracis* (*n* = 16) from heifers fed with different fiber sources.

	BS group	OH group	*p*-value
Final body weight (kg)	257.30 ± 8.182	241.24 ± 2.607	0.071
*M. longissimus* thoracis (kg)	5.59 ± 0.378	5.21 ± 0.467	0.550
Proximal composition			
Moisture (g/100 g)	73.12 ± 0.405	72.76 ± 0.443	0.560
Crude protein (g/100 g)	22.56 ± 0.213	22.95 ± 0.233	0.254
Crude fat (g/100 g)	2.87 ± 0.510	3.06 ± 0.587	0.816
Technological properties			
pH	5.59 ± 0.014	5.55 ± 0.036	0.405
L*	33.16 ± 1.569	32.16 ± 2.171	0.723
a*	13.94 ± 1.359	15.60 ± 2.166	0.545
b*	12.92 ± 1.114	16.53 ± 2.201	0.194
Fatty acid profile (g/100 g)			
C10:0	0.04 ± 0.00	0.05 ± 0.01	0.433
C12:0	0.09 ± 0.01	0.07 ± 0.00	0.025
C14:0	3.31 ± 0.17	2.88 ± 0.12	0.062
C14:1 n-5	0.64 ± 0.03	0.58 ± 0.07	0.503
C15:0	0.41 ± 0.03	0.30 ± 0.03	0.027
C16:0	26.00 ± 0.89	25.68 ± 0.68	0.777
C16:1 n-7	3.09 ± 0.06	3.05 ± 0.14	0.816
C17:0	1.07 ± 0.89	0.97 ± 0.07	0.229
C17:1	0.76 ± 0.09	0.65 ± 0.06	0.310
C18:0	15.36 ± 0.42	14.58 ± 0.64	0.346
C18:1 n-7	1.48 ± 0.32	1.82 ± 0.15	0.332
C18:1 n-9	37.11 ± 1.12	38.84 ± 1.05	0.283
C18:2 n-6	6.13 ± 1.18	6.18 ± 0.75	0.909
C18:3 n-3	0.35 ± 0.06	0.30 ± 0.04	0.511
C18:3 n-4	0.08 ± 0.01	0.08 ± 0.01	0.699
C18:3 n-6	0.10 ± 0.01	0.10 ± 0.01	0.909
C20:0	0.09 ± 0.01	0.09 ± 0.01	0.867
C20:1 n-9	0.24 ± 0.02	0.27 ± 0.02	0.372
C20:2 n-6	0.13 ± 0.02	0.12 ± 0.01	0.665
C20:3 n-6	0.42 ± 0.09	0.50 ± 0.10	0.566
C20:4 n-3	0.03 ± 0.01	0.03 ± 0.01	0.861
C20:4 n-6	1.58 ± 0.37	1.61 ± 0.28	0.937
C20:5 n-3	0.19 ± 0.05	0.17 ± 0.04	0.838
C22:0	0.02 ± 0.00	0.01 ± 0.00	0.004
C22:4 n-6	0.21 ± 0.04	0.23 ± 0.03	0.774
C22:5 n-3	0.46 ± 0.12	0.41 ± 0.10	0.734
C22:6 n-3	0.07 ± 0.02	0.05 ± 0.01	0.602
C24:1 n-9	0.02 ± 0.00	0.02 ± 0.00	0.590
SFA	46.41 ± 1.42	44.56 ± 1.26	0.350
MUFA	43.34 ± 1.25	45.24 ± 0.96	0.246
PUFA	10.25 ± 1.85	10.20 ± 1.32	0.983
n-3	1.15 ± 0.62	1.02 ± 0.20	0.675
n-6	8.44 ± 1.67	8.62 ± 1.14	0.929
n-6/ n-3	8.98 ± 0.69	8.52 ± 0.78	0.554

BS, Barley Straw; OH, Oat hay; MUFA, Monounsaturated fatty acids; PUFA, Polyunsaturated fatty acids. Data are presented as mean value ± standard error means.

Twenty-four volatile compounds were identified, most belonging to the alcohol group (Table 6). Nevertheless, in both groups regarding individual percentage abundance of volatile compounds, the most important compound was nonanal, belonging to the aldehyde group, followed by 2-Methyl-3-octanol corresponding to the alcohol group. Most of the volatile compounds detected exhibited a similar pattern (*p* > 0.05) between both experimental groups. However, certain trends (*p* < 0.1) were observed in some of these compounds. The 2-phenyl-1-ethanol, belonging to the alcohol group, showed a slightly higher content in the cooked MLT of the BS group than that of the OH group. This trend was similar to that described for 1,2,3-trimethylbenzene, which increased in the cooked MLT of the BS group.

**Table 6:** Aroma volatile (expressed as individual percentage abundance of volatile compounds) given off by cooked *M. longissimus thoracis* from heifers (*n* = 16) fed with two experimental diets.

Volatile compounds (%)	BS group	OH group	*p-*value
*Alcohols*			
2-Phenyl-1-ethanol	1.69 ± 0.61	0.46 ± 0.06	0.053
3-Ethyl-4-heptanol	6.27 ± 0.25	6.10 ± 0.23	0.626
2-Methyl-3-octanol	8.18 ± 0.41	8.65 ± 0.84	0.644
2.6-Dimethyl-3-heptanol	5.76 ± 0.27	5.79 ± 0.25	0.938
2-Methyl-3-octanol	8.67 ± 0.18	8.35 ± 0.25	0.335
1-Pentanol	0.90 ± 0.35	0.49 ± 0.08	0.255
1-Hexanol	0.14 ± 0.05	0.13 ± 0.05	0.849
4-Heptanol	4.38 ± 0.16	4.41 ± 0.35	0.950
1-Octanol	3.40 ± 0.42	2.62 ± 0.28	0.142
1-Octen-3-ol	1.06 ± 0.18	1.67 ± 0.50	0.304
1-Octen-4-ol	1.78 ± 0.18	1.73 ± 0.19	0.837
Aldehydes			
Hexanal	7.08 ± 3.67	5.94 ± 0.92	0.752
Octanal	4.53 ± 2.15	1.75 ± 0.21	0.191
Nonanal	12.47 ± 2.55	8.83 ± 1.48	0.227
Furans			
Furfural (2-Furaldehyd)	2.68 ± 1.95	0.72 ± 0.29	0.304
2-Pentylfuran	1.91 ± 0.92	1.59 ± 0.23	0.724
Homocyclic compounds			
1-Methyl-3-propylbenzene (Isobutyltoluene)	5.69 ± 0.65	5.30 ± 0.75	0.706
Ethylbenzene (Methyltoluene)	2.40 ± 1.35	1.01 ± 0.33	0.306
Benzenol	1.08 ± 0.62	0.39 ± 0.05	0.260
Benzaldehyde (benzene carbaldehyde)	6.13 ± 1.93	2.86 ± 0.42	0.101
Propylbenzene (1-Phenylpropane)	3.41 ± 1.65	2.99 ± 0.80	0.816
1,2,3-Trimethylbenzene	1.45 ± 0.12	1.11 ± 0.10	0.050
1,2,4-Trimethylbenzene (Pseudocumene)	1.09 ± 0.23	0.86 ± 0.09	0.342
*Sulfur compounds*			
Carbon disulfide (methanedithione)	6.89 ± 3.15	6.54 ± 2.00	0.924

1-Pentanol. BS, Barley Straw; OH, Oat hay. Data are presented as mean value ± standard error means.

### 3.3 Principal component analysis (PCA) and interpretation using biplot graphics

The PCA was performed for all variables assessed to obtain a reduced number of variables that explain the variability of the samples. The application of PCA has facilitated the analysis and similarities among groups since the number of variables has been reduced. Kaiser Rule was applied to select the number of PCs in both cases as the eigenvalue was higher than 1. The PCA was run on the database array of all parameters assessed in the current research. Four PCAs were selected as they explained about 75.06% of the variability. Each presented a cumulative proportion of 27.28, 25.95, 13.93, and 7.90%. It asserts that most of the variation observed is related to the impact of the dietary treatments. The PC1 was positively linked with the thickening region and negatively correlated with linoleic acid (C18:2 n-6). The PC2 was positively correlated with oleic acid (C18:1 n-9) and negatively associated with C22:5 n-3. The PC3 was positively related to C20:3 n-3 and negatively associated with yellowness (b*). Finally, the PC4 was positively linked with heneicosanoic acid (C20:0) and negatively related with 1-Hexanol.

**Figure 3:**
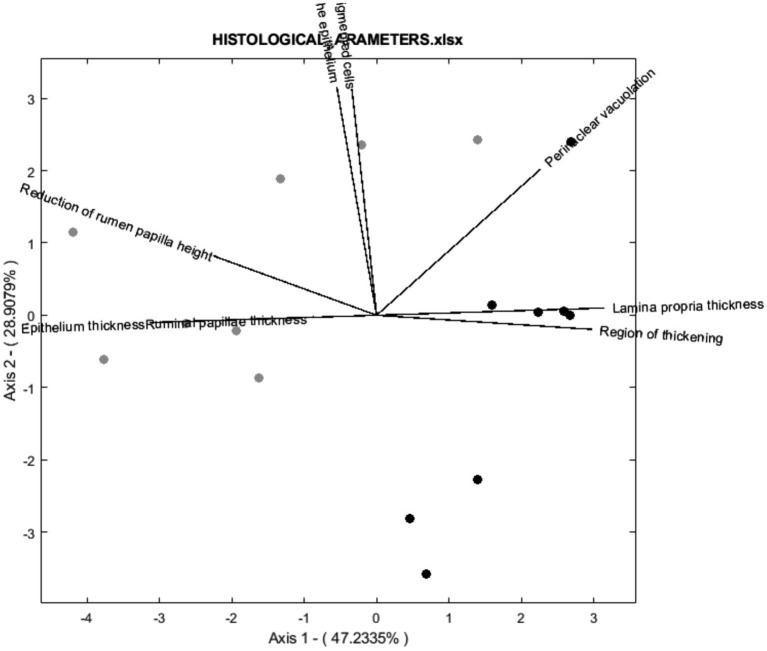
Vector spatial projection of the histological parameters evaluated from HJ-Biplot. Gray points refer to the Barley straw group (BS). Black points refer to the Oat hay group (OH).

**Figure 4:**
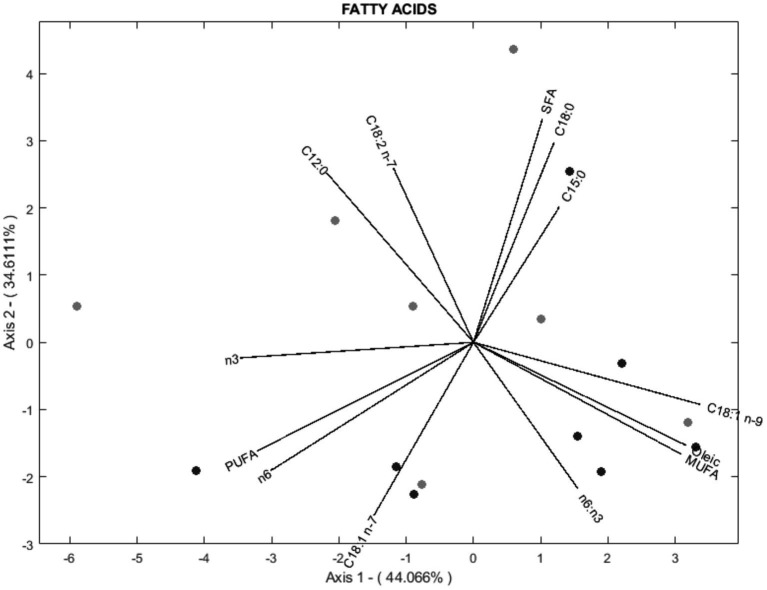
Vector spatial projection of the fatty acids evaluated from HJ-Biplot. Gray points refer to the Barley straw group (BS). Black points refer to the Oat hay group (OH).

**Figure 5:**
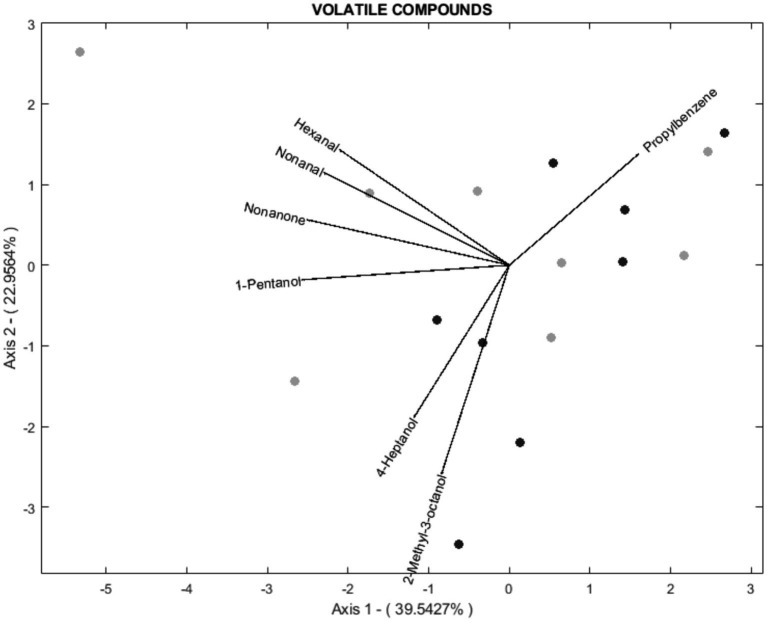
Vector spatial projection of the volatile compounds evaluated from HJ-Biplot. Gray points refer to the Barley straw group (BS). Black points refer to the Oat hay group (OH).

According to the biplot diagram, the degree of correlation can be interpreted as a function of the angle between lines and the size of the arrows. This method was chosen because it achieves the same goodness of fit for rows and columns (17). In this way, each variable’s magnitude of importance can be evaluated using the distance to the origin and the angle concerning others. For readability, 3 biplots were performed for histological parameters (Figure 3), fatty acids (Figure 4), and volatile compounds (Figure 5). As shown in Figure 3, the samples from the BS group were allocated in the first and fourth quadrants, while the most of OH samples were allocated in the second quadrants. The biplot diagram demonstrated several correlations between the studied variables. Ruminal papilla thickness and epithelium thickness were closely related. Similarly, the integrity of the epithelium showed a stronger correlation with the presence of hyperpigmented cells. Nevertheless, an inverse correlation was shown between the lamina of the above parameters and the lamina propria thickness. These two variables tended to be independent of the integrity of the epithelium and the presence of hyperpigmented cells. As presented in Figure 4, the variables related to SFA lead to the characterization of the BS group (upper quadrants of biplot), while MUFA and PUFA serve to define OH samples (lower quadrants of biplot). The biplot diagram showed a negative relationship between an isomer of oleic acid (C18:1 n-7) and the pentadecanoic acid (C15:0), this last one was negatively related to the ratio of n-6/n-3. It has been described as a close relationship between SFA, C18:0, and C15:0, similar to those observed between oleic acid and MUFA. Finally, the biplot corresponding to the volatile acid compounds (Figure 5) was not well defined, as the OH samples and the BS samples did not show a defined pattern in the quadrants.

## 4 Discussion

### 4.1 Ruminal histology findings

In the current research, most of the continuous variables assessed in the rumen were not significantly affected by the dietary treatment. Although no significant differences were observed between the experimental treatments, the animals in the BS group had a higher percentage of papilla height reduction. To our knowledge, the available literature studying the rumen histology of heifers fed with different forage sources is scarce, and most of them are related to the ratio of forage/concentrate (5, 8, 23). In this sense, Jiang et al. (26) proposed that changes in papillae height are more evident when dietary concentrate increases, which leads to an increase in ruminal propionate and butyrate concentration. To ensure pH homeostasis, it is necessary to increase the absorption of the acids produced, which leads to an increase in the height and width of the papillae. Besides, a reduction in papillae size is indicative of ruminal damage. Similarly, increased epithelial size has been related to the presence of inflammatory processes or adaptation after damage (6, 8, 9). According to those described above, the numerical results of papillae height of this research suggest a higher degree of rumen damage in the BS group. These findings seem to be confirmed by the results of the categorical ruminal variables.

The BS group exhibited a higher proportion of ruminal epithelium damage (with grades 2 and 3) compared to the OH group. The loss of differentiated cellular structure in the epithelium was more pronounced in the BS group, along with a greater presence of clefts in the papillae. Additionally, heifers from the BS group showed a significantly higher percentage of samples classified as grade 3 for hyperpigmented cells, similar to the findings for perinuclear vacuolation. Previous studies (6, 9, 27) have reported that disruption in epithelial integrity, increased vessel diameter, presence of eosinophilic content, and elevated presence of cytoplasmic vacuoles are indicative of inflammatory processes that may compromise the ruminal barrier, leading to the development of SARA. Although our observations suggest the onset of inflammatory processes, we did not find signs compatible with SARA in this study. Steele et al. (27) noted that ruminants have a high potential to adapt rumen histology to dietary changes, but this adaptability depends on the diet’s nutritional content and the duration of feeding. If an animal cannot adequately adapt, rumen lesions may develop, potentially leading to SARA symptoms. Based on our results and the biplot distribution, it appears that the ruminal adaptation capacity of the BS-fed animals was decreased. Although an improvement in the final weight of the animals could be expected if the rumen condition were improved, our study showed no differences in animal weight according to the source of fiber included in the diet. These findings are consistent with those described by Madruga et al. (11), who found no differences when a diet rich in forage was supplied to beef cattle compared to a diet containing OH, similar to those reported by Costa-Roura et al. (4). It is likely that other factors, such as feed intake, nutrient absorption, and even individual variation, contributed to the similar final body weights observed in both groups. Additionally, due to the duration of the study, it is possible that these changes were most evident at the rumen level, and that body weight was not significantly affected.

Given that the duration of the experiment and the chemical composition of the diets were identical for both groups, we can hypothesize that the histological differences observed may be attributed to the specific characteristics of the forages used. Gharechahi et al. (28) have shown that the physicochemical composition of forages, particularly their cellulose content, influences the microbial communities within the rumen. Gao et al. (29) found that replacing dietary BS with high-quality forages in cattle diets led to changes in the microbial population, improving fermentation pathways. These findings suggest potential differences in rumen microbial populations between the two diets, which may enhance ruminal status. However, further investigation of rumen microbial populations or pH is recommended to validate these results.

### 4.2 Carcass traits and meat quality

In the current research final body and MLT weights were not affected by the forage diet, which is similar to those described by Antúnez-Tort et al. (30) in post-weaning cattle when comparing alfalfa hay and BS. Costa-Roura et al. (4) described that the inclusion of OH did not compromise the performance of Holstein bulls compared to BS-based diets. Similar findings were reported in sheep fed with two experimental diets containing OH and BS (16). In the same line, Madruga et al. (11) found no significant differences in final body weight or dressing percentage when comparing diets rich in high-quality forages, such as alfalfa, to those containing BS. The lack of differences could be explained by the fact that both dietary treatments had the same energy content and NDF. Increasing the energy content and NDF of the diets could lead to a reduction in feed intake by increasing ruminant time, which subsequently results in a reduction of final body weight (17).

The nutritive content plays an essential role in the global quality of meat (31). In the current research, the chemical composition was unaffected by the fiber source which is similar to those described in beef (11), cattle (29), steers (32), and sheep (16) feed with comparative forage sources. Meat pH has an important influence on meat quality, as it affects color, water-holding capacity, and tenderness (11, 32). No differences were found between the two treatments for pH value, with both values being within the range described as optimal (5.4–5.8) for beef (11). These results are consistent with those reported by previous studies (11, 29, 32) evaluating different sources of forage in the diet. The variations in pH observed after 24 h can be attributed to the glycogen content of the muscle at slaughter, which is influenced by the stress experienced during pre-slaughter handling. Despite the animals being subjected to the same processing conditions, including handling procedures, it would be reasonable to anticipate uniform pH values.

Meat color is one of the most important attributes in purchase decisions (33). In the current research, no differences were detected in any parameters of meat color between the two experimental diets. These color changes are typically associated with post-mortem acidification. Therefore, the lack of differences in the final pH values could explain the consistent color results obtained. Our findings are consistent with previous research by Madruga et al. (11), who also found no significant differences in meat color when using alfalfa hay in beef diets. This alignment with previous studies supports the idea that dietary variations may not always result in observable differences in meat color, particularly when post-mortem pH levels remain consistent. Similarly, the findings reported by Amett et al. (34) also support our findings, suggesting that dietary factors may have minimal impact on meat color when pH remains unchanged. Contrarily, Ducket et al. (35) found darker-colored meat when steers were finished with forages than concentrates. The impact of dietary supplementation with various forages on meat color has been a topic of debate, with potential associations to both the type of forage used and the method of its preservation. Previous research suggests that including green forages in the diet may lead to higher carotenoid content than dry forages (e.g., hay), which could influence meat color deposition (36). However, even when evaluating the same forage type, variations in carotenoid and tocopherol content may arise due to differences in maturity stage (36), potentially contributing to discrepancies observed across studies.

Meat fatty acid composition is strongly related to the benefits for humans (24). While it is true that the SFA content was numerically higher in the BS group compared to the OH group, this difference was not statistically significant. This indicates that, within the parameters of our study, the variation in total SFA content between the two groups may not be robust enough to draw definitive conclusions about the health implications of the meat produced from these dietary treatments. However, it is noteworthy that certain SFAs, such as C12:0, C14:0, C15:0, and C22:0, which are considered less beneficial for human health (2, 24), were found in higher concentrations in the BS-fed heifers. Despite these findings, it would be premature to conclusively state that meat from OH-fed heifers is healthier based solely on our results. The observed trends suggest potential benefits of using OH over BS, but these preliminary results should be interpreted with caution. Longer-term studies involving a larger sample size are necessary to validate these findings and to better understand the impact of different forage types on meat fatty acid composition and overall health implications. Further research should also consider additional factors such as feed intake, metabolic responses, and the interaction of various dietary components to provide a comprehensive assessment of meat quality and its health effects. It has been described that the tannin content of forage could affect ruminal microbes by blocking ruminal biohydrogenation, resulting in a lower content of certain SFA (29). Moreover, ruminal microbial communities could be affected by cellulose content present in the forage, resulting in a modulation of fatty acid metabolism (28, 29). The prevalence of one particular microbial species compared to other species may impact ruminal metabolism, which could affect muscle fatty acid deposition (11). For example, Liu et al. (37) reported an increased abundance of *Prevotella* and *Fibrobacter* as forage content increased as these microorganisms participate in the metabolism of certain nutrients and promote their absorption. We speculated that the two hypotheses proposed could explain the results obtained. However, according to Van Elswyk et al. (38), the impact of forage feeding on the fatty acid profile of beef is not straightforward to generalize. Variations in the fatty acid profile may be influenced by factors such as the animal breed, the specific muscle analyzed, and even the type of forage used. Therefore, additional research is necessary to conclusively demonstrate the potential benefits of incorporating OH into heifer diets on the fatty acid composition of the meat.

Raw meat is recognized for its weak odor; nonetheless, it represents a matrix rich in non-volatile precursors of volatile compounds that contribute to the flavor of meat products. One significant source in the production of volatiles is the fat tissue, which suffers modifications leading to the production of several reactive substances, including acids, alcohols, aldehydes, and ketones (39). In the current research, aldehydes were the most abundant volatile compounds aligning with previous reports for cooked beef (39). The aldehyde content in meat is related to lipid oxidation processes and higher levels of unsaturated fatty acids (UFA) could be responsible for the increased aldehyde content. Given the minimal variation among both experimental diets, it was expected that there would be no significant differences in aromatic content between the two diets as observed in the biplot. Slight differences were found in only two compounds: -phenyl-1-ethanol (an alcohol), and 1,2,3-trimethylbenzene (a homocyclic compound) both of which were higher in the cooked MLT of the BS group compared to the OH group. Alcohol exerts an effect on the flavor of the meat. Alcohols can influence the flavor of meat, and low concentrations of 2-phenyl-1-ethanol in cooked meat have been associated with a fruity aroma. The MLT of the OH group recorded lower values of this compound, suggesting a possible improvement in odor attributes; however, a sensory analysis would be necessary to confirm these results. Studies have demonstrated that feeding practices and animal breed can affect the volatile compound content (38, 39). Many studies have focused on comparing the ratio of concentrate versus forage, showing controversial results (40, 41). To our knowledge, variations in volatile content have not been evaluated as a function of the type of forage included in the diet. Therefore, further studies are needed to confirm our findings.

### 4.3 Principal component analysis

Principal component analysis (PCA) aims to minimize the dimensionality of variables while keeping the highest variance in the rest of the dimensions using an orthogonal processing (18). A large number of variables were evaluated in this research, including rumen health and meat quality. The analysis demonstrates that variability could be explained by four components (PCs) as it presented a 75.06% variability. Hence, 75.06% of the total variance in the 77 variables assessed could be condensed into four new variables. A biplot diagram is a valuable way to identify not only the variables but also well as the relationships among them (18). Biplot diagram allows an instant visual identification of the variables that are correlated with each other, and their direction. Those diagrams revealed that the meat produced by the OH diet could be discriminated from that of the BH diet. Most of the samples from the meat of heifers fed OH are in quadrants 1 and 4, where MUFA, oleic acid, ratio n-6: n-3, C18:1 n-7, perinuclear vacuolation, lamina propria thickness, region of thickening are located. The meat of heifers fed with BS was primarily allocated to the second and third quadrants regarding histological parameters and to the upper quadrants regarding fatty acids. However, the distribution of the sample according to aromatic compounds was not well defined. These statistical findings support the previously mentioned differences among the experimental fiber diets, indicating that dietary changes to include different fiber sources (OH or BS) could affect ruminal health status and produce meat with varying quality parameters.

## 5 Conclusion

The inclusion of OH in heifer diets did not affect final body weight, meat composition, or technological properties compared to BS. Although certain saturated fatty acids (SFA) were lower in OH- meat, suggesting potential health benefits, this does not imply overall meat health as only specific fatty acids were affected. The provision of OH in the diet appeared to have positive effects on rumen health status compared to heifers receiving BS. While these improvements may indicate a potential reduction in metabolic diseases such as SARA, it is essential to acknowledge that direct indicators of SARA were not measured in this study. PCA and biplots illustrated relationships between diets and variables, supporting OH’s potential in sustainable farming. Nevertheless, to validate and expand upon these results, further studies are warranted, including an evaluation of ruminal microbiota and pH in these animals. Such investigations will provide a more comprehensive understanding of the potential benefits of incorporating OH into heifer diets and its implications for animal health and product quality.

## Data availability statement

The original contributions presented in the study are included in the article/supplementary material, further inquiries can be directed to the corresponding author.

## Ethics statement

The animal studies were approved by Institutional Animal Care and Use Committee of the Agrarian Technological Institute of Castilla y León. The studies were conducted in accordance with the local legislation (42) and institutional requirements. Written informed consent was obtained from the owners for the participation of their animals in this study.

## Author contributions

AB-D: Formal analysis, Investigation, Software, Writing – original draft, Writing – review & editing. AS-G: Conceptualization, Formal analysis, Methodology, Supervision, Validation, Writing – original draft, Writing – review & editing. JG-G: Conceptualization, Funding acquisition, Methodology, Project administration, Resources, Writing – review & editing. CV: Formal analysis, Investigation, Writing – review & editing. ED: Investigation, Writing – review & editing. RB: Conceptualization, Investigation, Writing – review & editing. LG-G: Investigation, Writing – review & editing. PV-G: Formal analysis, Supervision, Writing – review & editing.
